# Influence of Calcium Crosslinker Form on Alginate Hydrogel Properties

**DOI:** 10.3390/gels11110885

**Published:** 2025-11-04

**Authors:** Solomiia Kapatsila, Roman Taras, Diana Varchuk, Nataliia Nosova, Serhii Varvarenko, Volodymyr Samaryk

**Affiliations:** Department of Organic Chemistry, Lviv Polytechnic National University, 12 Bandera Str., 79013 Lviv, Ukraine; solomiia.m.kapatsila@lpnu.ua (S.K.); roman.s.taras@lpnu.ua (R.T.); diana.varchuk.mkhtkhd.2024@lpnu.ua (D.V.); nataliia.h.nosova@lpnu.ua (N.N.); serhii.m.varvarenko@lpnu.ua (S.V.)

**Keywords:** alginate, polyacrylic acid, swelling, gel-fraction, mesh size

## Abstract

Alginate hydrogels are attractive for biomedical applications and drug delivery due to their biocompatibility and biodegradability. However, calcium-crosslinked alginates often exhibit only moderate absorption properties compared with synthetic hydrogels. This study examined how the form of calcium ion delivery affects the mechanical, swelling, and morphological characteristics of calcium-crosslinked alginate hydrogels. We prepared four alginate hydrogel samples in which Ca^2+^ was introduced on different polyacrylate polymer carriers, and a reference hydrogel crosslinked with calcium citrate. All samples were characterized by equilibrium swelling, gel fraction determination, and rheological frequency-sweep measurements. Also, the average mesh size was estimated using two independent theoretical approaches. Hydrogels prepared with calcium salt of polyacrylic acid (PAA) exhibited higher mechanical strength and higher water swelling than the citrate-crosslinked reference. Calculated mean mesh sizes for the citrate system ranged from 58 to 221 nm, whereas high-molecular-weight crosslinked systems showed a broader distribution (68–708 nm). These results demonstrate that the form of Ca^2+^ introduction significantly influences network architecture and functional properties and indicates that tuning the carrier form of calcium provides a practical route to design swelling, mesh size, and mechanical behavior of alginate-based hydrogels for specific biomedical or delivery applications.

## 1. Introduction

In recent years, the use of hydrogel materials has grown significantly. Both synthetic and natural hydrogels are widely utilized [1,2,3,4,5]. In medical practice, there is a trend toward favoring hydrogels made from biocompatible, non-toxic polymers of natural origin, such as polysaccharides (sodium alginate (SA), chitosan, cellulose). As a result, many studies have focused on the behavior of these materials, particularly on alginate hydrogels [6,7,8]. Special attention is given to SA-based hydrogels, which form under mild conditions [9,10,11]. The process of SA hydrogel formation with calcium ions is straightforward and is described by the “egg-box model.” According to this model, one calcium ion binds to two pairs of guluronic acid residues through ion coordination [12,13]. These “boxes” can assemble into multi-tiered structures (dimers, tetramers, octamers) of various lengths. Their formation is influenced by both the distribution and the ratio of mannuronic to guluronic acid residues, as well as the molar concentration of calcium ions in solution. It is worth noting that such supramolecular structures are rarely ideal, as the presence of sodium ions and traces of other metals introduces structural defects [14,15].

Controlling how the hydrogel absorbs liquids is essential because it influences the material’s ability to function—such as retaining or gradually releasing active substances, controlling humidity, and removing exudate—and also impacts its mechanical stability and shape during swelling [16]. This absorption trait enables a variety of uses: high and selective absorption—for medical dressings and hygiene products; controlled swelling and liquid release—for drug delivery systems and agricultural matrices; and controlled swelling, which forms the basis for sensors, actuating components, and regulated porosity in tissue engineering [17,18]. Therefore, the mechanical and absorption characteristics of alginate hydrogels can be effectively managed by adjusting the type and concentration of ionic crosslinkers [19,20,21]. For instance, crosslinking with a 0.1 M iron (III) chloride solution, compared to a 0.1 M calcium chloride solution under the same conditions, increases hydrogel stiffness (Young’s modulus rising from 71.5 kPa to 252 kPa). Meanwhile, the equilibrium degree of swelling decreases from roughly 43 g of water per g of dried alginate-calcium hydrogel to 31 g of water per g of dried alginate-calcium hydrogel [22].

An alternative approach to forming alginate hydrogels is covalent crosslinking, particularly through polysaccharide modification [23]. Carboxyl functional groups can react with diamine molecules, such as adipic acid dihydrazide, hexamethylenediamine, and lysine-derived diamine. More commonly, however, covalent crosslinking requires pre-oxidation of the SA to form alginate dialdehyde, which can react with amino-containing polymers to form stable imine bonds [24]. Such hydrogels can be further modified by introducing boronate ester bonds, thereby creating a double dynamic covalent mesh [25,26]. Compared to ionically crosslinked alginate, covalently crosslinked hydrogels exhibit a lower swelling capacity [27,28]. While the ionically crosslinked alginate hydrogels have a higher viscous dissipation (G″ ≈ 10 Pa) and undergo adhesive failure at strain amplitudes above 100%, the covalently crosslinked hydrogels behave more elastically (G′ ≈ 100 Pa, G″ ≈ 1 Pa) and fail cohesively at lower strains (γ_0_ ≈ 60–140% depending on the concentration of the crosslinker). Furthermore, the critical energy release rate increases with calcium concentration in ionically crosslinked gels (0.01–0.023 Jm^−2^ for 10–15 mM Ca^2+^), but remains relatively constant in covalently crosslinked gels (≈0.04–0.07 Jm^−2^), suggesting more stable crosslinking [29].

In alginate hydrogels, where the calcium salt of ethylenebis-(oxy-ethylenenitrilo) tetraacetic acid (chelation agent) acts as a crosslinker, the formation of the polymer network proceeds more slowly, resulting in a more structurally homogeneous network. These hydrogels demonstrate a swelling capacity of up to 250% in physiological fluid at pH 6.8. Samples containing 10 mmol of calcium ions show high stiffness and elasticity (78.08 g and 10.73 g, respectively). In terms of mechanical properties, the elastic modulus of such hydrogels does not exceed 0.7–0.8 kPa at calcium concentrations of 10–20 mmol, indicating the formation of a dense polymer network with low porosity and a low degradation rate [30,31]. Increasing calcium chloride concentration as a crosslinker significantly affects the morphology and swelling capacity. At higher concentrations, excessive ionic interactions compromise structural homogeneity and mechanical integrity, whereas lower concentrations of calcium chloride (0.40–0.60%) slow gelation and weaken crosslinking, producing hydrogels with lower rigidity but increased absorption capacity, albeit with reduced structural stability [32].

Water-soluble calcium salts have several common drawbacks, including difficulty in precisely controlling the crosslinking process and the inability to achieve high homogeneity in the calcium-alginate network [11,22,33]. To solve this issue, either introduce calcium-ion dissolution inhibitors (in the form of chelating compounds) or substitute the calcium compound carrier. In particular, the use of glucono-δ-lactone in combination with calcium carbonate facilitates controlled crosslinking, preventing excessively rapid, heterogeneous crosslinking, which is typical of aqueous calcium chloride solutions. Such a slow-gelling system leads to more homogeneous network formation (gelation time varies from 10 to 30 min, depending on glucono-δ-lactone concentration). Alginate hydrogels retain stable mechanical properties after 28 days of storage. Their structural integrity values are higher than those of lactone-free control samples [34].

These alginate hydrogels, in which calcium polyacrylate acts as the crosslinker, form a homogeneous polymer network through ionic interactions between calcium ions and SA carboxyl groups, as well as additional physical interactions between the PAA and alginate macromolecular chains [35,36]. When combined with gelatin (which is not crosslinked by calcium ions), these hydrogels exhibit a high gel fraction (GF) of up to 96.6% and maintain good mechanical integrity under compressive loads, reaching up to 17.04 kPa. Depending on the ratio of calcium ions to carboxyl groups, the degree of swelling ranges from 62.9 to 574.1 g water per g polymer, which is directly affected by the degree of calcium substitution in the crosslinker. These alginate hydrogels retain sufficient elasticity and structural stability even after exudate absorption, making them suitable for drug-delivery applications [37,38]. Using glycerol to inhibit salt dissolution and to disperse calcium salts is a more promising approach than introducing lactone: in glycerol, both inorganic and polymeric calcium salts remain dispersed rather than dissolving, whereas lactone typically requires dissolution in water. Moreover, this method of calcium introduction increases medium viscosity, slowing ion diffusion and thus promoting more homogeneous network crosslinking. It also reduces water activity, thereby affecting swelling [39,40,41].

The crosslinking density of alginate hydrogels plays an essential role in microstructural studies of these materials. The density and distribution of crosslinks determine the gel’s mesh size and mechanical rigidity, which, in turn, influence diffusion processes [20,42]. For example, 1% SA samples crosslinked with 100 mmol CaCl_2_ show a mesh cell size of 19.8 ± 2.8 nm and a low elastic modulus of 3.91 kPa, whereas 2% SA samples (200 mmol CaCl_2_) exhibit a much smaller mesh size (4.3 ± 0.4 nm) and a higher elastic modulus (18.4 kPa). Understanding how to control crosslinking density—by adjusting crosslinker concentration or the polymer content of the matrix—is essential for optimizing hydrogel formulations for various applications [43].

Despite numerous studies on the use of calcium chloride, calcium phosphate, and calcium carbonate as crosslinkers for SA, a systematic comparison of low-molecular-weight, organic, and polymeric forms of calcium introduced as glycerol dispersions (without additional inhibitors) is still lacking. Such a comparison is necessary both to design alginate-hydrogel properties intentionally and to control absorption–release behavior in drug delivery or artificial matrix applications. Pore size is a key parameter for absorption–release processes because, during swelling, the polymer chains relax and straighten, increasing the network volume and allowing molecules to penetrate the mesh [3,44,45]. Flory and Rehner developed a theoretical framework to calculate the crosslink density of three-dimensional polymer networks, and subsequent authors extended these ideas to estimate pore (mesh) size [19,46,47,48]. That theory relies on simplifying assumptions—notably that the network is effectively homogeneous and that loops and dangling chains are negligible—which limit its applicability in realistic, heterogeneous gels [49]. As shown earlier, higher crosslink density corresponds to smaller mesh sizes. An increase in the calcium chloride content as a crosslinker in the alginate hydrogel from 100 mmol to 200 mmol significantly reduces the hydrogel mesh initial size: a hydrogel of 1% SA with 100 mmol calcium chloride has a mesh size of 19.8 ± 2.8 nm, while the same hydrogel with twice the crosslinker content has a mesh size of 16.3 ± 0.3 nm. Thus, a higher crosslinking density, obtained by increasing the concentrations of both SA and calcium chloride, is observed in hydrogels with a pore size of 4.3 ± 0.4 nm, which also exhibit a shear modulus of 18.4 kPa. When submerged in an aqueous medium, the mesh size increases—for a 1% SA hydrogel containing 100 mmol of calcium chloride, the cell size increases from 19.8 nm to 240.2 nm after 10 days [43]. Similar trends are seen in semi-synthetic networks: raising the crosslinker concentration reduced mesh size from hundreds to a few tens of nanometers (from 450 nm to 30 nm), and hydrogels with higher glutaraldehyde content show noticeably smaller pores than those with lower amounts, confirming that pore size can be tuned readily by adjusting crosslinker concentration [50].

The authors argue that the crosslinker–carrier structure strongly influences node formation, network homogeneity, and the efficiency of crosslinking SA into a three-dimensional network. Introducing polymeric calcium salts yields a distinct hydrogel morphology and considerably changes the material’s mechanical and absorption properties.

## 2. Results and Discussion

**Figure 1:**
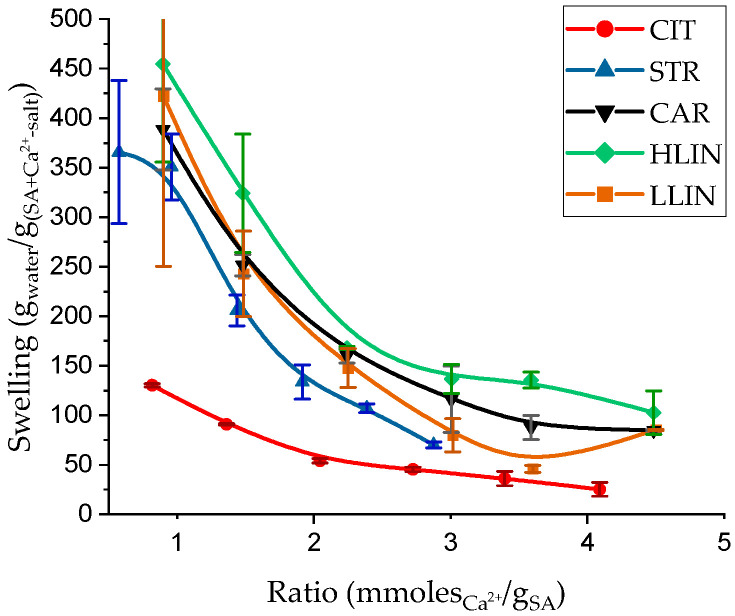
Dependence of the equilibrium swelling degree on the ratio of calcium ions to the SA mass in hydrogel samples. CIT—SA crosslinked with calcium citrate, STR—SA crosslinked with rarely structured calcium polyacrylate, CAR—SA crosslinked with carbomer-940 calcium salt, HLIN—SA crosslinked with higher molecular weight calcium polyacrylate, LLIN—SA crosslinked with a lower molecular weight calcium polyacrylate.

Key characteristics of hydrogel materials that determine their potential applications are their water absorption capacity and physical–mechanical properties. To investigate the effect of the structuring agent, we analyzed both the equilibrium swelling degree and the mechanical behavior of the hydrogels. Figure 1 shows the correlation between the equilibrium swelling degree and the ratio of structuring agent (mmol) to SA (g) for five hydrogel series.

The data presented show that increased network structuring leads to decreased equilibrium swelling of the hydrogels. The nature of the counter-ion also strongly affects the swelling of the polymer network. At a ratio of 0.5 moles of Ca^2+^ to 1.0 g of SA, CIT (SA crosslinked with calcium citrate) samples absorb up to 125 g/g, whereas hydrogels crosslinked with PAA calcium salts reach 300–450 g/g. The increase is mainly due to replacing the low-molecular-weight crosslinker (calcium citrate) with a polymeric one. Thus, the equilibrium swelling of STR (SA crosslinked with rarely structured calcium polyacrylate) samples is 2.4 times higher than CIT. In contrast, LLIN (SA crosslinked with a lower molecular weight calcium polyacrylate) or HLIN (SA crosslinked with a higher molecular weight calcium polyacrylate) hydrogels show 3.6 times higher values. Moreover, the molecular weight of linear PAA affects swelling: HLIN samples consistently exhibit 10–120% higher equilibrium swelling than LLIN at the sameratios. These results demonstrate that the structure of PAA macromolecules significantly affects the swelling properties of alginate hydrogels.

To further understand how these structural differences influence hydrogel performance, the physical and mechanical properties of the same samples were investigated.

**Figure 2:**
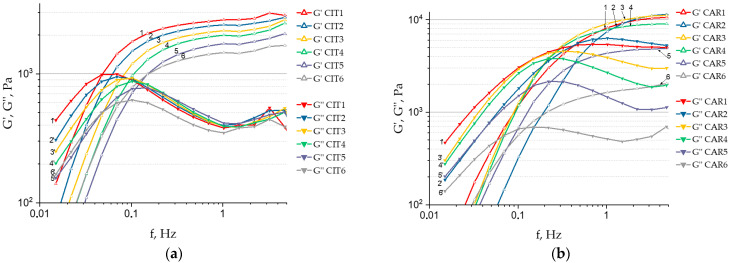
Frequency sweep for CIT samples (**a**), and CAR samples (**b**). Marks 1–6 denote the curves of the accumulated modulus (G′) for the samples of the corresponding number, marks 1′–6′ denote the curves of the loss modulus (G′) for the samples of the corresponding number.

Figure 2 depicts characteristic dependencies of the accumulated (G′) and lost (G″) moduli on the applied frequency for the studied objects.

**Figure 3:**
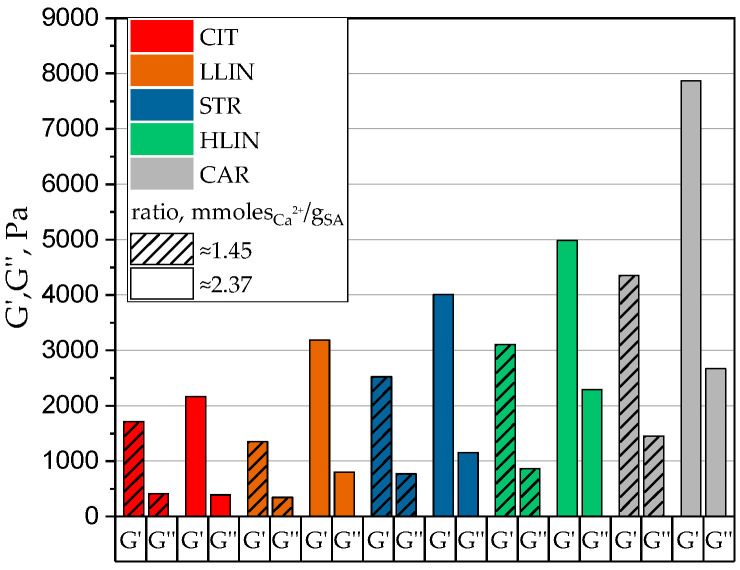
Bar diagram of G′ and G′ (at 1 Hz) values obtained from oscillation tests on hydrogels for two different ratios of calcium ions to SA.

The presented dependencies demonstrate the predominance of elastic properties within a specific frequency range (G′ > G″), a characteristic of physically crosslinked hydrogels. In addition, a plateau of G′ values is observed at higher frequencies (0.2–15 Hz), indicating network stability under rapid deformation. As expected, the physical and mechanical characteristics of hydrogels (G′ and G″) significantly depend on the ratio between calcium ions and SA. An increased amount of the structuring agent promotes the formation of a more developed polymer network due to the increased number of intermolecular bonds. Comparison of frequency sweep curves in Figure 2 indicates that the amount of calcium ions alone does not fully determine hydrogel mechanics. The form of the counter-ion associated with calcium also significantly influences the physical and mechanical properties. To illustrate this effect, Figure 3 shows G′ and G″ values at a frequency of 1 Hz for hydrogel samples with two different ratios of calcium ions to SA.

This diagram demonstrates that when PAA, in any form (except LLIN at a ratio of 1.45), is used as the counter-ion to calcium, the G′ and G″ moduli of the hydrogels are significantly higher at both crosslinker-to-SA ratios compared to those obtained using calcium citrate. The most significant increase (3–4 fold) is achieved with SA crosslinked with carbomer-940 calcium salt (CAR), and high-molecular-weight linear PAA (HLIN) is used as the crosslinker.

Analysis of the physical and mechanical properties indicated that using PAA as a calcium carrier alters the hydrogel morphology. This is supported by comparing the G′/G″ ratios for hydrogel samples structured with different calcium salts. For calcium citrate hydrogels (CIT), the values of G′ are higher than G″ by 6–8 times (at a frequency of 1 Hz, within the range of 0.5–4.0 mmol Ca^2+^ per gram of SA). For LLIN or HLIN hydrogels, this correlation ranges from 1.5 to 2.0. This data reflects a significant increase in plasticity when using calcium salts of PAA as crosslinkers.

**Figure 4:**
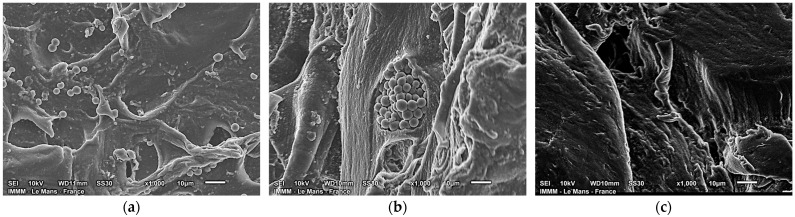
SEM micrographs of the Au-sputtered cryo-fractured surfaces of the lyophilized hydrogel samples: (**a**)—STR sample, (**b**)—inclusion of microspheres of rarely structured PAA in the STR sample, (**c**)—LLIN sample.

Among the PAA-containing systems, STR hydrogels show the weakest effect: under the same conditions, the G′/G″ ratio is 3.6–4.0, which is closest to that of CIT hydrogels. This suggests that the rarely structured PAA plays a limited role in the development of the crosslinked hydrogel network. This conclusion is confirmed by SEM images of cryo-fractured STR hydrogels (Figure 4a). For clarity, Figure 4b highlights an unhomogenized inclusion of dispersed, rarely structured PAA calcium salt embedded within the hydrogel network.

As shown in the micrograph fragment, the dispersed salt particles form regular spheres with diameters of 1.5–2.0 μm under the hydrogel formation conditions. These spherical particles largely occupy the bulk of the STR hydrogels (see Figure 4a). We ascribe this morphology to the fact that the rarely structured PAA—already present as a preformed polymer network—does not thoroughly homogenize during gelation but becomes incorporated into the alginate matrix as heterogeneous inclusions. The observed morphology—alginate matrices containing dispersed inclusions of PAA hydrogels—significantly increases the equilibrium swelling (Figure 1), likely due to the additional free volume and hydrophilic domains introduced by the inclusions. In contrast, the mechanical reinforcement is comparatively modest compared with that of hydrogels crosslinked with other calcium salts, suggesting that the inclusions primarily enhance water uptake rather than network stiffness. When crosslinkers of different structures were used, no phase separation or release of PAA into a distinct phase was observed. For instance, Figure 4c shows an SEM image of the cryo-fractured surface of the LLIN hydrogel sample, which shows no such inclusions.

**Figure 5:**
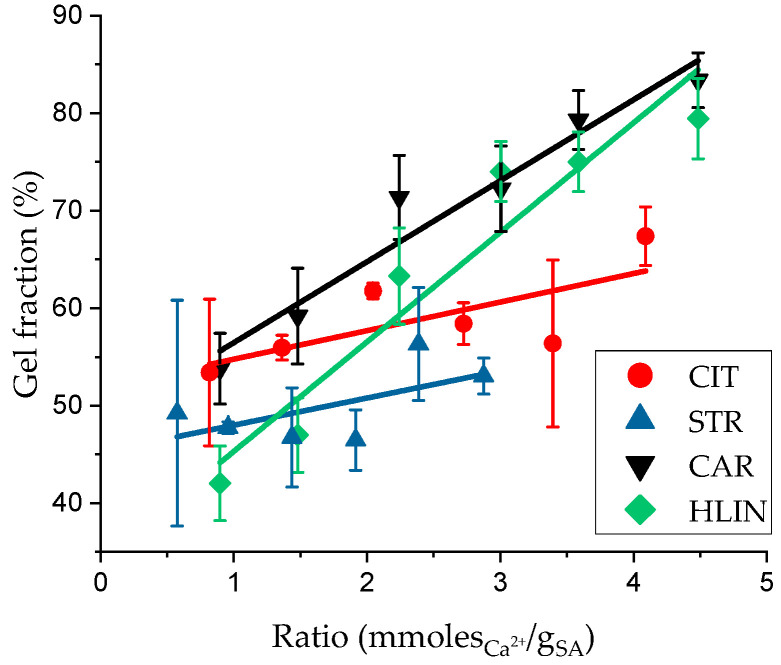
Gel fraction of hydrogel samples as a function of the ratio between calcium ions and SA.

One key hydrogel characteristic is GF content. Figure 5 shows the dependence of GF content on the ratio between the crosslinker and the polymer. As shown, the GF varies in the range of 50–80% depending on the sample composition. At the same time, the use of high-molecular-weight compounds as counter-ions to calcium does not exert a decisive influence on the GF, suggesting that the introduction of PAA into the system does not substantially alter the mechanism of SA network formation.

However, specific trends can be observed. For hydrogels structured with calcium citrate, increasing the Ca^2+^/SA ratio does not significantly increase GF content. A similar behavior is observed for STR samples. These patterns can be attributed to the limited impact of the citrate anion and rarely structured PAA on the structuring process. The citrate anion, as a low-molecular-weight compound, contributes minimally to the network structuring, while the macromolecules of rarely structured PAA, as shown in the SEM images in Figure 4a,b, act primarily as a source of calcium ions rather than as structural components of the network.

In contrast, the HLIN and CAR samples exhibit a proportional increase in the GF with increasing calcium ion content. These results indicated that PAA with this macrochain structure participates in forming the hydrogel network. This conclusion aligns with the described effects on swelling and mechanical properties.

**Figure 6:**
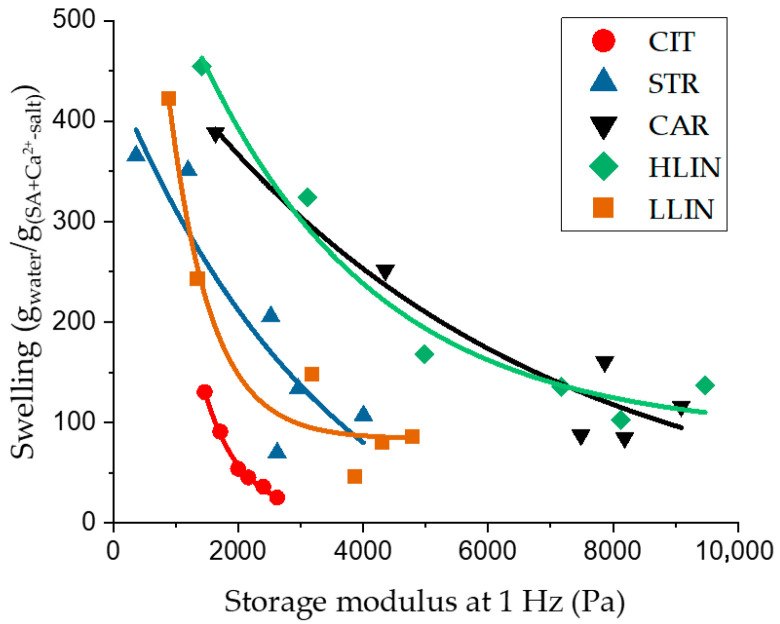
Dependence of equilibrium swelling in water on the elastic modulus at 1 Hz (oscillation test).

The effect of the counter-ion structure of the ionic crosslinker can also be characterized by assessing the density of network crosslinks and the mesh size. The latter parameter can be determined using two approaches: the equilibrium swelling theory (based on the equilibrium swelling data) and the theory of rubber-like elasticity (based on the rheological measurements). Figure 6 presents the dependence curves of the equilibrium swelling values and the elastic modulus of the hydrogel samples. The presence and consistency of the correlation between these parameters for each hydrogel series suggest that the network structuring parameters can be reliably estimated using both theories, as described in the works [51,52].

The results of these calculations obtained by Equations (3)–(8) are summarized in Table 1.

**Figure 7:**
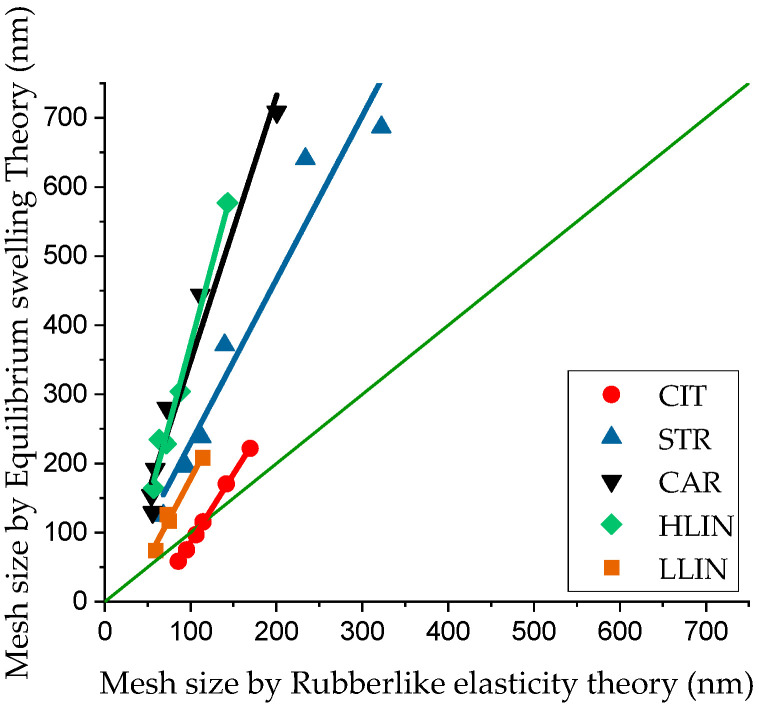
Scatter diagram of cell size values for five series of alginate hydrogel samples calculated using two theoretical approaches.

Analysis of the presented data reveals the following observations. The average cell sizes estimated by the two theoretical approaches do not coincide. Figure 7 shows a scatter diagram comparing the hydrogel cell size calculated by the two methods. In both theories, the mesh size is obtained from Equation (3) after determination of the molecular weight between crosslinks, M_c_—the polymer molecular weight between two network nodes—using Equation (4) (by equilibrium swelling theory) or (5) (by rubber-like elasticity theory). For the equilibrium swelling theory, M_c_ is calculated from the equilibrium swelling degree and the experimentally determined volume fraction of network-forming polymers υ_2_. For the rubber-like elasticity theory, the key parameter is the shear modulus G, defined as the value at which the storage modulus G′ is independent of oscillation frequency.

**Figure 8:**
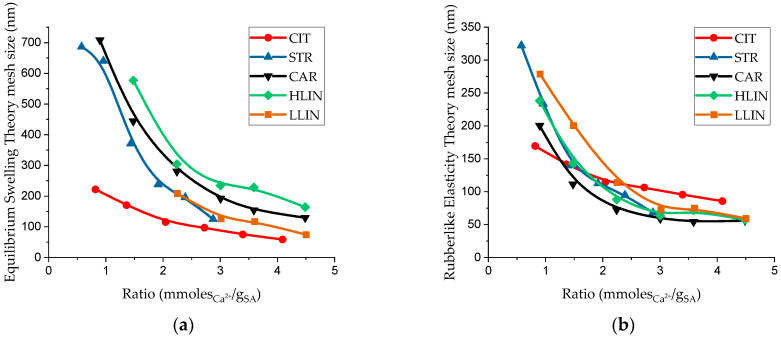
Curves of dependence of the mesh size of hydrogels ((**a**)—calculated according to the theory of equilibrium swelling, (**b**)—calculated according to the theory of rubber-like elasticity) on the ratio between calcium ions and SA.

This diagram shows that values obtained from different theories are most consistent (closest to the diagonal) for CIT samples. In contrast, a significant discrepancy is observed for samples based on PAA calcium salts. Across all sample series, the mesh size calculated using the equilibrium swelling theory is consistently higher than that obtained from the rubber-like elasticity theory. The differences can be explained by the different nature of the original data of the methods: the first evaluates the average volumetric state of the polymer network and to some extent includes the contribution of defects not involved in the grid of chains and unevenly stitched areas, and the second measures the active grid through the shear modulus, so it shows smaller effective cell sizes. For samples based on polyacrylate salts, additional ionic associates, differences in the molecular weight of the chains, and physical interactions can increase water loss, which overestimates the indicators of equilibrium swelling, but do not participate in the transmission of elastic stress. Figure 8 shows the dependence of the average mesh size on the calcium-to-SA ratio.

As expected, these dependencies show a decrease in cell size with increasing crosslinker-to-polymer ratio, attributed to the greater number of crosslinking points formed. The theories used to estimate this parameter predict different effects of this ratio on the mesh size. According to the equilibrium swelling theory, which deploys equilibrium swelling degree as a key experimental parameter, a significant increase in mesh size is predicted at all ratios. In contrast, the rubber-like elasticity theory predicts a substantial increase in mesh size only at low crosslinker-to-polymer ratios within the studied range.

The incorporation of PAA macromolecules in any of the studied forms noticeably affects their mechanical properties at low ratios and the equilibrium swelling values across all studied ratios compared with CIT samples. It is unambiguously evident that an increase in the molecular weight between crosslinks, and consequently in the average mesh size of the hydrogel network, leads to a higher equilibrium swelling degree and a reduction in the physical and mechanical properties.

The incorporation of polyelectrolytes, such as PAA, into the polymer network promotes network expansion via electrostatic intersegmental repulsion between ionized carboxyl groups. Furthermore, PAA macromolecules possess higher water absorption capacities than SA due to the formation of strong hydrogen bonds. As a result, the enhanced swelling caused by PAA can lead to an overestimation of the molecular weight between cross-links and significant discrepancies in the calculated mesh size. Therefore, mesh size estimates derived from the rubber-like elasticity theory should be considered more accurate and reliable under these conditions.

## 3. Conclusions

The properties of alginate hydrogels formed by ionic crosslinking depend significantly not only on the nature of the metal ion (for example, Ca^2+^, Zn^2+^, Al^3+^) but also on the nature of the counterion, which acts as a structuring agent. As part of this study, we showed that the fundamental characteristics of alginate hydrogels—degree of swelling and physical and mechanical properties—are determined not only by the type of metal but also by the chemical nature and macromolecular structure of the counterion to calcium ions. In particular, a comparison of calcium citrate-structured hydrogels (CIT) and calcium polyacrylate-structured hydrogels demonstrated a dramatic change in properties when the low-molecular-weight counterion was replaced with the polymer counterion. The use of PAA increases the equilibrium swelling degree by approximately 4–5 times, due to the duplication of calcium ions within the hydrogel structure. At the same time, there is a noticeable increase in mechanical characteristics, in particular, the actual modulus of elasticity (detailed quantitative values are given in the Section 2). The degree and nature of this influence depend significantly on the PAA macromolecular structure, molecular weight, conformation, and degree of branching. Mechanistically, this effect is associated with changes in the morphology of the polymer grid: the partial inclusion of PAA in the alginate network and the formation of additional ionic associates contribute to strengthening matrix connectivity and altering pore structure. As a result, in systems with PAA, the volumetric capacity for water absorption increases (which is reflected in a greater equilibrium degree of swelling). At the same time, the active (loadable) mesh, which governs elastic properties, is enhanced by additional interchain connections and associates. Consequently, cell size estimates obtained by methods based on equilibrium swelling and rubber-like elasticity can systematically differ due to the presence of weakly bound fractions and morphology changes. Considering the obtained results, it can be argued that controlling the structure of the counter-ion relative to calcium ions is an effective tool for fine-tuning the degree of swelling, mechanical properties, and morphology of alginate hydrogels. This is of practical importance in the development of materials for the food and medical industries, where requirements for water absorption, mechanical resistance, and morphology are primary. For further research, it is recommended to study in more detail the influence of PAA molecular weight and structure on the long-term stability, reducing properties, and biocompatibility of hydrogels, and to systematically compare quantitative estimates of cell size obtained by different theoretical approaches with morphological data.

## 4. Materials and Methods

### 4.1. Materials

SA (CAS Number: 9005-38-3, Sigma-Aldrich, St. Louis, MO, USA) was used without further purification. Calcium oxide (CAS Number: 1305-78-8, Sigma-Aldrich), glycerol (CAS Number: 56-81-5, Sigma-Aldrich). Calcium citrate tetrahydrate (CAS Number: 5785-44-4, Sigma-Aldrich). Polyacrylic acid with molecular weight 3–5 kDa, 50 wt.% solution in water (CAS Number: 9003-01-4, ThermoFisher Scientific, Waltham, MA, USA). Polyacrylic acid with molecular weight 240 kDa, 25 wt.% solution in water (CAS Number: 9003-01-4, ThermoFisher Scientific). Carbomer-940 (CAS Number: 151687-96-6, Lipotec Srl, Bologna, Italy). Acetone (CAS Number: 67-64-1, Sigma-Aldrich). Rarely structured polyacrylic acid was obtained according to [53] methodic. Distilled water, with a specific electrical conductivity of 5–7 µS·cm^−1^ and pH = 6.5 ± 0.1, was used.

### 4.2. Crosslinker Synthesis

CaO was added with vigorous stirring to a 5% aqueous solution of a linear, rarely structured, or carbomer-940 polyacrylic acid. On each 1.0 mole of CaO, there were added 2.3 moles of polyacrylic acid. The mixture was stirred until heat generation ceased, after which the top transparent layer was allowed to settle and decant. The additional calcium salt was washed with propanone, then dried at 60 °C to a constant weight.

### 4.3. Hydrogel Synthesis

SA powder is dissolved in water with stirring for 24 h to obtain a solution with a mass concentration of 2.60%. The weight of each calcium salt is added to glycerin to obtain a dispersion with a calcium salt content of 10.0–18.0%. In every sample, the alginate concentration remains unchanged, and the calcium salt dispersions are diluted with glycerin. The mass compositions of all hydrogel samples are shown in Table 2; each sample contains 2.20% SA, and the rest is water.

**Figure 9:**
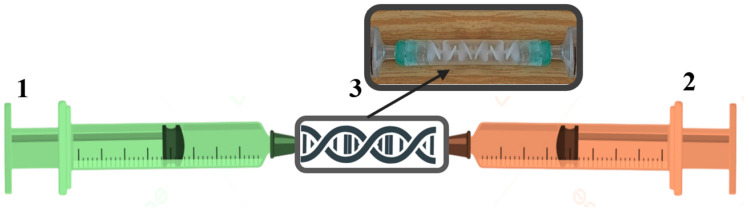
Schematic representation of the installation for creating calcium alginate hydrogels: 1—syringe with polymer solution, 2—syringe with calcium salt dispersion, 3—connector with mixing nozzle.

Figure 9 depicts a setup for homogenizing polymer and crosslinker solutions. In one 10 mL syringe, 6.00 g of SA solution is drawn; in another, 1.00 g of calcium salt dispersion is drawn. The syringes are connected to the mixing nozzle via a connector, and the mixture is pressed by the pistons with reciprocating movements until a homogeneous mixture is achieved within 10 s.

After that, the mixture is pressed onto a Petri dish with a diameter of 50 mm, and the formed hydrogel disk has a height of 4–5 mm. The sample is sealed overnight at room temperature to fully form the hydrogel structure.

### 4.4. Equilibrium Swelling in Water

The swelling ratio of the alginate hydrogel was determined gravimetrically. The sample was immersed in 30 cm^3^ of distilled water and incubated at room temperature for 72 h. The sample mass was measured after this time by decanting the liquid medium and blotting the sample with filter paper.

Swelling of the alginate hydrogel per mass of gel-forming polymers was calculated according to the equation:

(1) $$SR=\frac{m_{1}-m_{0}}{\left(\omega_{SA}+\omega_{Ca-salt}\right)\cdot m_{0}},$$

where *m*_1_ is the mass of the swollen sample, g; *m*_0_—initial mass of the sample, g; *ω_SA_*—SA content in the sample, parts per unit; *ω_Ca-salt_*—calcium salt content in the sample, parts per unit.

### 4.5. Calcium Salt Density

The sample was weighed on an analytical balance in air, then weighed in a 3D-printed hydrostatic weighing unit [54]. Hexane was chosen as the medium in which the substances do not dissolve.

The following equation determined the density of the sample:

(2) $$\rho_{Ca-salt}=\rho_{hex}^{t}\cdot \frac{m_{air}}{m_{air}-m_{hex}},$$

where $\rho_{hex}^{t}$ is the density of hexane at the measurement temperature, g·cm^−3^; *m_air_*—the mass of the sample in air, g; *m_hex_*—the mass of the same sample in hexane, g

### 4.6. Mesh Size Evaluation

The following equation determines the pore size:

(3) $$\zeta =\frac{l\cdot \sqrt{\frac{2\cdot M_{c}}{M_{r}}}\cdot \sqrt{C_{n}}}{\sqrt[{3}]{\upsilon_{2}}},$$

where *M_r_* is the molecular weight of repeating unit, for alginate hydrogel—390.1 g·mol^−1^ [43]; *M_c_* is the molecular weight between crosslinks, g·mol^−1^; *C_n_* is the characteristic ratio reflecting the stiffness or flexibility of the polymer chain, *C_n_* = 0.021·*M_polymer_* + 17.95 (where *M_polymer_* is the polymer molecular weight in kDa, in this case used SA has *M_polymer_* = 538 kDa); *l* is the length of the carbon–carbon bond in the monomer, for alginate units—0.515 nm [50]; *υ*_2_—the volume fraction of network components in the swollen sample.

The following equation calculates the molecular weight between the crosslinks for each hydrogel according to the equilibrium swelling theory:

(4) $$M_{c}^{s}=\frac{\rho_{p}}{n_{s}},$$

where $\rho_{p}$ is the density of dried polymer mesh washed from the sol fraction experimentally determined by hydrostatic weighing, g·cm^−3^, and n_s_ is the concentration of mesh chains, mol·cm^−3^.

Also, the molecular weight between crosslinks can be calculated based on the values of the shear modulus (G, Pa) using the following equation from the rubber-like elasticity theory:

(5) $$M_{c}^{r}=\frac{c_{p}\cdot R\cdot T}{G},$$

where *c_p_* is the concentration of polymer (SA) in the tested hydrogel, kg·m^−3^; *R* is the gas constant, 8.314 m^3^·Pa·mol^−1^·K^−1^; and *T* is the temperature, K.

The concentration of network chains represents the number of active network chains fully included in an ideal network per unit volume and is determined by the Flory–Rehner equation:

(6) $$n_{s}=-\frac{\ln\left(1-\upsilon_{2}\right)+\upsilon_{2}+\chi \cdot \upsilon_{2}^{2}}{V_{0}\cdot \left(\sqrt[{3}]{\upsilon_{2}}-2\cdot \frac{\upsilon_{2}}{f}\right)},$$

where *χ* is the interaction constant between the polymer network and the solvent (water), *V*_0_ is the molar volume of water (18.0 cm^3^·mol^−1^), and *f* denotes the functionality of a network node, that is, the average number of polymer chains converging in one node, in this case *f* = 4.

To solve Equation (6), the volume fraction of network components υ_2_ is calculated according to the following equation:

(7) $$\upsilon_{2}=\frac{\omega_{SA}\cdot \rho_{SA}+\omega_{C}\cdot \rho_{C}}{\omega_{SA}\cdot \rho_{SA}+\omega_{C}\cdot \rho_{C}+\omega_{G}\cdot \rho_{G}+\omega_{W}\cdot \rho_{W}},$$

where *ω* is the mass fraction of the component in the swollen hydrogel, *ρ* is the density of each element separately, g·cm^−3^.

The precise value of the Flory–Huggins parameter is difficult to determine for multi-component systems, so this study uses approximate calculations, which are obtained based on the volume fraction of network components according to the following equation:

(8) $$\chi =\frac{\ln\left(1-\upsilon_{2}\right)+\upsilon_{2}}{\upsilon_{2}^{2}}.$$

Calculated values of the Flory–Huggins parameter are within the −0.510 to −0.501 range and were used to interpret the system’s swelling behavior.

### 4.7. Gel Fraction

A weighed sample of the hydrogel is placed in a pre-weighed, pencil-signed filter bag, which is then tied with a thread. Each 20 samples are placed in a mesh in a magnetically stirred beaker and poured with 2.5 dm^3^ of distilled water. The system is heated to 30–35 °C and left under stirring for 24 h, during which the water is changed to fresh water every 8 h. After that, the bags are removed from the mesh, untied and dried in a drying cabinet to a constant mass.

The GF in the hydrogel is calculated according to the following equation:

(9) $$GF=\frac{m_{2}-m_{p}}{\left(\omega_{SA}+\omega_{Ca-salt}\right)\cdot m_{0}}\cdot 100\%,$$

where *m*_0_ is the initial mass of the sample, g; *m*_2_—the mass of the sample in the bag after washing, g; *m_p_*—the mass of the filter bag, g.

### 4.8. Dynamic Mechanical Test

A stress-controlled rheometer (HAAKE MARS iQ, ThermoFisher Scientific, Waltham, MA, USA) was used for rheological characterization. A 25 mm parallel-plate geometry was used, with the gap between the plates adjusted to the sample height. The prerequisite was that the upper geometry always touched the sample surface with a normal force of 0.15 N. Frequency sweep tests were conducted (at τ_0_ = 500 Pa (which was found inside the LVER in the previously made amplitude sweep) and f = 0.015 Hz–15 Hz). Raw stress–strain signals were analyzed using HHAKE RheoWin 4.94 software. All rheological measurements were conducted at 20 °C. Stress–strain data affected by cohesive gel failure or slippage at the gel-plate interface (adhesive failure) were excluded from data analysis.

### 4.9. Scanning Electron Microscopy

The Scanning Electron Microscope used is a JSM-6510LV (JEOL, Tokyo, Japan). The samples were swollen twice, frozen in liquid nitrogen, lyophilized (the materials were lyophilized in a Christ freeze-dryer alpha 2–4 LSC at −85 °C and 0.37 mbar), sectioned to visualize the hydrogel morphology, and covered with a golden film before examination. Images of the hydrogels were analyzed using ImageJ Software Version 1.54.

### 4.10. Statistical Analysis

All experiments were conducted in triplicate, and results were reported as mean ± SD. Mean values were compared using an independent-samples Student’s *t*-test, with *p*-values < 0.05 considered statistically significant.

## Figures and Tables

**Table 1:** The results of calculating the polymer network mesh size according to two theories.

Sample Name	ρ_p_, g⋅cm^−3^	Equilibrium Swelling Theory				Rubber-like Elasticity Theory		
		υ_2_, %	n_s_ 10^5^, mol cm^−3^	$M_{c}^{s}$, kDa	ζ, nm	G, kPa	$M_{c}^{r}$, kDa	ζ, nm
CIT1	1.564	2.91	16.3	9.6	59	2.61	20.54	86
CIT2	1.516	2.33	11.1	13.6	75	2.44	22.01	95
CIT3	1.702	1.97	8.37	20.3	97	2.20	24.40	106
CIT4	1.721	1.71	6.56	26.3	116	2.08	25.75	115
CIT5	1.521	1.17	3.44	44.2	170	1.76	30.51	142
CIT6	1.294	0.87	2.10	61.6	222	1.49	35.96	169
STR1	1.507	1.51	5.34	28.2	125	6.38	8.41	68
STR2	1.674	1.08	3.02	55.4	196	4.12	13.01	95
STR3	1.578	0.89	2.19	72.2	238	3.32	16.15	113
STR4	1.597	0.61	1.17	136.8	371	2.77	19.38	140
STR5	1.539	0.38	0.52	295.6	641	1.36	39.38	234
STR6	1.584	0.36	0.48	329.1	687	0.74	72.48	322
CAR1	1.382	1.41	4.76	29.0	129	9.90	5.42	56
CAR2	1.730	1.34	4.36	39.6	154	10.87	4.93	54
CAR3	1.511	1.05	2.89	52.3	192	10.83	4.95	59
CAR4	1.628	0.79	1.77	91.9	280	8.77	6.11	72
CAR5	1.559	0.52	0.89	175.5	444	4.85	11.06	111
CAR6	1.531	0.35	0.45	340.8	708	1.97	27.27	200
HLIN1	1.444	1.18	3.50	41.3	164	10.74	4.99	57
HLIN2	1.562	0.92	2.30	67.9	229	8.07	6.64	72
HLIN3	1.564	0.90	2.22	70.4	234	10.36	5.17	64
HLIN4	1.677	0.74	1.61	104.2	304	6.12	8.76	88
HLIN5	1.509	0.41	0.60	253.1	577	3.44	15.59	143
HLIN6	*	0.30	0.35	*	*	1.54	34.89	238
LLIN1	1.333	2.22	10.3	13.0	74	6.53	8.21	59
LLIN2	1.197	1.45	4.96	24.1	117	5.46	9.83	75
LLIN3	1.174	1.35	4.38	26.8	126	6.05	8.86	73
LLIN4	1.074	0.85	2.01	53.5	209	3.35	16.02	114
LLIN5	*	0.54	0.94	*	*	1.46	36.68	201
LLIN6	*	0.32	0.40	*	*	1.06	50.46	279

*—failed to measure.

**Table 2:** The hydrogel’s composition.

Code	CIT1	CIT2	CIT3	CIT4	CIT5	CIT6
Glycerin, %	12.79	13.04	13.29	13.54	13.79	13.99
Calcium citrate salt, %	1.50	1.25	1.00	0.75	0.50	0.30
Code	STR1	STR2	STR3	STR4	STR5	STR6
Glycerin, %	12.79	13.04	13.29	13.54	13.79	13.99
Rarely structured PAA calcium salt, %	1.50	1.25	1.00	0.75	0.50	0.30
Code	CAR1	CAR2	CAR3	CAR4	CAR5	CAR6
Glycerin, %	11.7	12.22	12.55	12.99	13.43	13.77
Carbomer-940 calcium salt, %	2.59	2.07	1.73	1.29	0.85	0.52
Code	HLIN1	HLIN2	HLIN3	HLIN4	HLIN5	HLIN6
Glycerin, %	12.13	12.56	12.84	13.21	13.57	13.85
High-molecular-weight linear PAA calcium salt, %	2.16	1.73	1.45	1.08	0.71	0.43
Code	LLIN1	LLIN2	LLIN3	LLIN4	LLIN5	LLIN6
Glycerin, %	12.00	12.46	12.75	13.14	13.53	13.83
Low-molecular-weight linear PAA calcium salt, %	2.29	1.83	1.53	1.14	0.75	0.46

## Data Availability

All data and materials are available upon request from the corresponding author. The data are not publicly available due to ongoing research using a part of the data.

## Abbreviations

The following abbreviations are used in this manuscript:

GF	Gel fraction
SA	Sodium alginate
PAA	Poly(acrylic acid)
